# Patients' perceptions and experiences of the prevention of hospital-acquired thrombosis: a qualitative study

**DOI:** 10.1136/bmjopen-2016-013839

**Published:** 2016-12-13

**Authors:** Patricia N Apenteng, David Fitzmaurice, Ian Litchfield, Sian Harrison, Carl Heneghan, Alison Ward, Sheila Greenfield

**Affiliations:** 1 Institute of Applied Health Research, College of Medical and Dental Sciences, University of Birmingham, Birmingham, UK; 2 Nuffield Department of Primary Care Health Sciences, University of Oxford, Oxford, UK

**Keywords:** venous thromboembolism, prophylaxis, patient education, QUALITATIVE RESEARCH, deep vein thrombosis, pulmonary embolism

## Abstract

**Objective:**

To examine patients' understanding of hospital-associated thrombosis, and their experiences of thromboprophylaxis.

**Design:**

Qualitative study using semi-structured interviews with 31 patients requiring venous thromboembolism (VTE) prophylaxis following a recent hospital admission. Interviews were audio-recorded, transcribed verbatim and analysed thematically using framework analysis.

**Setting:**

4 hospitals in Birmingham and Oxford.

**Results:**

All the participants received thromboprophylaxis following surgical procedures. Participants were aware of a risk of blood clots; however, they lacked a good understanding of VTE and its components. Experiences of VTE prophylaxis were characterised with good adherence to heparin injections and poor adherence to elastic compression stockings, largely due to perceived lack of clarity in guidance from health professionals. Participants had limited knowledge of the signs and symptoms of VTE and would value improved education on VTE.

**Conclusions:**

Findings suggest that patient education is often inadequate and impacts negatively on patients' involvement in VTE prevention. An enhanced patient education programme incorporating a consistent message on the appropriate use of elastic compression stockings and description of VTE symptoms is likely to optimise the effectiveness of the prevention of hospital-associated thrombosis. Physicians may use the results of this study to improve individual patient education.

**Strengths and limitations of this study**

- Mixed variety sample of patients requiring venous thromboembolism prophylaxis following a recent hospital admission.
- Interviews were audio recorded, transcribed verbatim and independently checked for accuracy.
- Data analysis was iterative and independent of the interviewing researcher.
- Over-representation of surgical patients.
- Participants were predominantly of white British ethnicity.

## Introduction

Patient involvement is an important aspect of the prevention of hospital-associated thrombosis (HAT) yet, to date, much of the focus on preventing HAT has been on health professionals' implementation of the venous thromboembolism (VTE) prevention strategy and associated outcomes, and there is little understanding of patients' perceptions and experiences of HAT prevention. HAT can occur up to 90 days postdischarge from hospital, and it is recognised that patients are at increased risk during this time with most cases of HAT occurring following discharge.^1^ With current trends towards enhanced recovery and early postsurgery hospital discharge, a significant proportion of hospitalised patients at risk of VTE are discharged with mechanical prophylaxis (usually antiembolism stockings) and/or pharmacological prophylaxis (usually self-administered injections of low molecular weight heparin).^2–5^ Therefore, a lot of responsibility falls on the patient with regard to appropriate use and adherence to VTE prophylaxis and recognition of possible VTE episodes for timely medical intervention.

VTE comprises the acute conditions deep vein thrombosis (DVT) and pulmonary embolism (PE); DVT occurs when a blood clot forms in the deep veins (usually lower legs) and PE is a potentially fatal complication which occurs when some or the entire clot detaches and travels to the lungs. A recent US study found that while patients were generally aware of the benefits of antithrombotic therapy, only 6 out of 12 interviewed patients had a clear understanding of DVT and PE.^6^ Also adherence has been identified as a problem with patients prescribed thromboprophylaxis both internationally and in the UK.^7–10^ It has been hypothesised that patients are not adequately educated about the rationale for thromboprophylaxis; however, this has not been fully elucidated and no qualitative research has explored HAT prevention from the patients' perspective. This qualitative study, embedded within a larger study exploring the prevention and knowledge of VTE (ExPeKT) aimed to explore patients’ awareness of VTE and their experiences VTE prophylaxis.^11^

## Methods

Face-to-face interviews were carried out with patients classed by hospital staff as being at high risk of developing VTE during a recent hospital admission. The rationale of using interviews was to allow detailed exploration of personal perceptions and individual experiences without the contamination of other participants' views. Participants were drawn from respondents to a survey conducted as part of the broader ExPeKT study.^11^ The survey was distributed to 868 inpatients assessed to be at high risk of VTE, recruited from medical, surgical and orthopaedic wards in two acute trusts in Oxford and Birmingham. Of these, 564 patients returned completed questionnaires and 238 confirmed they would be prepared to be interviewed.

Purposeful sampling^12^ was employed to select interview participants of maximum variety of age, gender, condition requiring hospital stay and site. This was to ensure that the sample reflected a varied range of patients to minimise the risk of the study being distorted to one perspective. A topic guide was developed through discussion with the research team, and comprised open-ended questions that drew reflections on patients' recent hospital admissions with particular reference to their understanding of VTE risk and their experiences of how this risk was assessed and managed (see online supplementary appendix 1).

10.1136/bmjopen-2016-013839.supp1 supplementary appendix

Data collection continued until theoretical saturation was attained.^13^ Semistructured interviews were conducted with a total of 31 patients and they all took place in the patients' homes. All participants provided informed consent prior to the interviews. The interviewer was a woman, a non-clinical researcher (PhD) trained in qualitative research; she was not known to participants prior to the study, and participants were made aware that she was conducting the interviews as part of her job. The interviews lasted between 10 and 45 min; all were audio recorded and transcribed verbatim. Verification of interview data was completed through triangulation with the corresponding survey responses to establish credibility and dependablity.^14^

### Analysis

PNA, IL and SG independently read through the same three interview transcripts to familiarise themselves with the interviews and identify emerging themes. They then met to compare, discuss and finalise themes for the coding frame.^15^ Based on this PNA subsequently coded the remaining interviews using NVivo software to manage the data which was analysed using framework analysis^16^ (see online supplementary appendix 2).

10.1136/bmjopen-2016-013839.supp2 supplementary appendix

## Results

### Participant characteristics

Participants' characteristics were extracted from patient questionnaires administered as part of the wider ExPeKT study^11^ (table 1). Of the 31 participants, 55% were men and ages ranged from 38 to 81 years with a mean age of 63. The majority (94%) were of white British ethnicity and 87% had received at minimum an ‘O level’ education or earned a professional qualification.

**Table 1:** Participants' characteristics

Variable	N=31	Per cent
Male, n (%)	17	54.8
Age groups (years)		
≤40	3	9.7
41–64	11	35.5
65–74	12	38.7
≥75	4	12.9
Not known	1	3.2
White British ethnicity	29	93.5
Highest level of education received		
O or A level	6	19.4
Degree	9	29.1
Professional/commercial	12	38.7
Not known/none	4	12.9
Admission		
Planned admission	27	87.1
Emergency admission	4	12.9
Length of hospital stay, number of days		
≤3	7	22.6
1–6	17	54.8
** **≥7	7	22.6
Condition requiring hospital admission		
Orthopaedic surgery	18	58.1
Gastrointestinal surgery	7*	22.6
Other surgery	6†	19.3
VTE prophylaxis		
Stockings only	5	16.1
Injectable prophylaxis only	2	6.5
Both stockings and injectable prophylaxis	24	77.4

*One was oncology related.

†Three were oncology related.

VTE, venous thromboembolism.

All the participants were surgical patients, 87% were planned admissions and the remainder were emergency admissions. In total, 58% underwent orthopaedic surgery (hip or knee replacement). All the participants received VTE prophylaxis, with 77% receiving both compression stockings and heparin injections.

**Figure 1:**
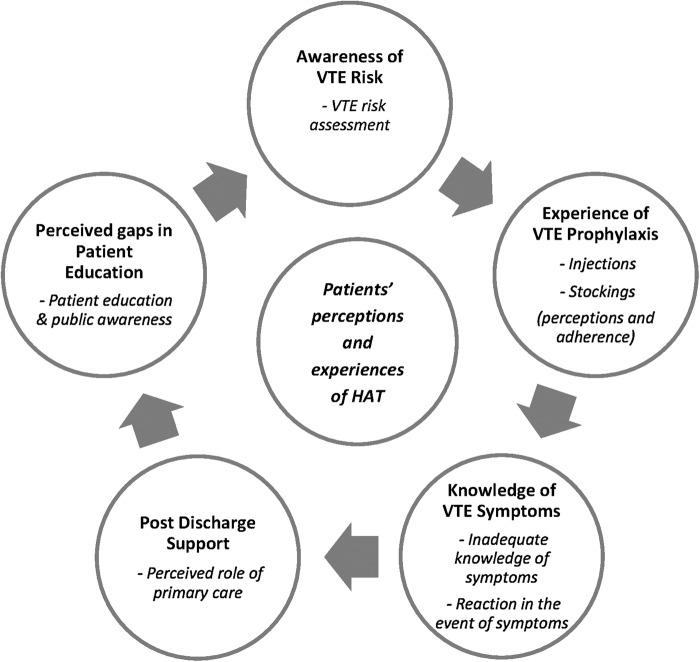
Themes and subthemes. HAT, hospital-associated thrombosis; VTE, venous thromboembolism.

Findings are presented in five main themes: awareness of VTE risk, experience of VTE prophylaxis, knowledge of VTE symptoms, postdischarge support and perceived gaps in patient education (figure 1).

### Awareness of VTE risk

Patients reported being aware of risk of blood clots associated with their recent hospital admission; though they did not refer specifically to the terms DVT or PE. In particular, patients booked for planned orthopaedic surgery described a presurgical assessment which entailed a discussion on all the risks relating to their surgery including risk of blood clots.

I just remember the general things that there is obviously a substantial risk of clotting and that they take certain precautions to ensure that they manage that sort of during and after the process. *Female aged 69, total hip replacement*

Yes they were very good. They covered all those points honestly, they did point out lower limb surgery, increased risk etc. Best thing to do, and that's come through repeatedly, is keep moving. *Male aged 56, total hip replacement*

However, information given to a patient in the preassessment for non-orthopaedic surgery seemed to miss out the emphasis on blood clots:

I mean I know when you have your pre-op they do your blood pressure and everything, yes. But at no time, in fact, were blood clots actually mentioned. *Female aged 68, stoma reversal*

Patients' source of awareness of VTE appeared to be mainly from information given in work up to the recent surgical admission or a previous surgery of themselves or a family member.

Q: Before you went into hospital were you aware of the risks of blood clots associated with being in hospital?

A: Yes, I think so, largely because, I'm having been in previously for various bits and pieces over my life, y’know, I was aware that they can happen but fortunately there's no family history so, not too concerned. And also everyone explains these things at each time, all the various risks that are associated. *Female aged 62, knee replacement*

*VTE risk assessment*: Patients appeared not to be aware of undergoing a VTE risk assessment and many presumed that the VTE prophylaxis was part of a general approach rather than a tailored treatment due to their being at high risk.

I had a pre-med, they checked my blood pressure and everything, but no I wasn't aware of being risk assessed for that particular condition (blood clots). *Female aged 63, ovarian cysts*

### Experience of VTE prophylaxis

Patients described their perceptions and experiences of heparin injections and compression stockings.

*Injections: perceptions and adherence*: Twenty-four out of 26 patients given heparin injections as part of their hospital admission had to self-administer the heparin injections at home postdischarge. Patients' narratives portrayed mixed views of having to self-inject; some reported they were not keen on self-administering the injections while others had no problem with it and did it routinely. Patients described the differing levels of guidance provided on injecting the chemoprophylaxis. Some received training by a nurse that included a demonstration and observation while others recalled being handed the injection set on discharge and instructed to maintain the course of injections. Despite these discrepancies all participants discharged with heparin injections reported completing the course of injections though some had help from an adult child or partner.

I mean I'm not particularly a squeamish kind of person, so I wasn't particularly nervous about it. I didn't like the idea when I first had to do them the first time, but, realising effectively that there isn't a choice, just get on and do it. So…y’know 6 o'clock every night the ol’ thing came out…I had quite a few bruises on my tummy…as time went on I suppose y’know, it did become more painful. But y’know, needs must, if that's what you've got to do, that's what you've got to do. *Female aged 62, knee replacement*

Generally patients understood that the injections were to prevent blood clots; however, two patients had limited understanding of the rationale for the injections and viewed them as part of the treatment of their condition, and did not necessarily associate them with VTE prevention.

Q: So what treatment did you receive, to prevent blood clots?

A: Now, was I given any pills, the week before. On that visit, we were given some pills…no I don't think before the operation I had anything specific for blood clots.

Q: Did you have anything whilst you were in hospital do you know?

A: I'm sure they were giving me something, they were giving me various pills, but again, they didn't necessarily tell me, as I recall, what it was for.

Q: What about when you left the hospital. Did they give you anything to bring home?

A: I don't think so; I mean I had the support stockings…

Q: Did you have any injections? Injections in the stomach?

A: Yes, ah, is that, was that, I'd forgotten that now. I was given these—I had to inject myself for, a month?

Q: Yes, now that is an anticoagulant

A: Right, perhaps I had forgotten that. *Male aged 63, total hip replacement*

*Elastic compression stockings: perceptions and adherence*: A total of 29 patients reported receiving elastic compression stockings during their hospital admission and described their experiences. Overall, there was inconsistency in the administration of the stockings and some participants described coming round from surgery to find the stockings on, with no explanations. Patients' narratives pointed to a lack of clarity on the use of stockings and few patients (7/29) wore them for prescribed length of time. Patients described multifaceted reasons for non-adherence; guidance on stockings use seemed to be rather potluck—very few were clear that they were to continue to wear them postdischarge and/or how long they were to wear them for.

Q: Did you wear the stockings all of time?

A: For the full length I was in there, yeah.

Q: And when you came out?

A: Didn't wear them when I came out.

Q: Were you supposed to?

A: Well no I don't think so…I didn't wear them when I came out no, no. Although they did give me four or five pairs. *Male aged 38, lump removed from right leg*

Some patients discontinued wearing stockings after a while due to discomfort, choosing to exercise and/or stay mobile in place of the stockings or a perception that their personal risk of VTE was low.

I carried on for a little while, but they made my legs worse. I felt they were too tight…My daughter in law is a physiotherapist- because I was so active, she thought I would be alright without sort of thing…so uncomfortable they bit into you around the top here leg and all that y'know. *Male aged 73, knee replacement*

Patients also reported contradictions in information received regarding stockings, with conflicting information from nurses, doctors and information leaflets. This made it difficult for patients to know the correct course of action, and one patient was told he no longer needed to wear the stockings once the injections were started.

The nurse said wear them for a fortnight which is what I did and then reading the leaflet afterwards it said keep wearing the stockings for after six weeks but I only wore them for a fortnight. *Male aged 69, hip replacement*

I suppose that's probably the most unclear part of the whole procedure. The injections were fine, I was quite happy with y'know, doing and administering it and that whole process. Obviously the stockings were worn in the hospital sort of continuously and then I think the information you get, the information sheets is wear of up to four weeks post op, but there was definitely conflicting advice from the nursing staff. Some of the nurses were, ‘well it's not that serious if you don't wear them’ others were ‘absolutely must wear them.’ So, y'know there was definitely conflicting advice. *Female aged 40, hip replacement revision*

Some of the patients who wore elastic compression stockings for the prescribed period (often 6 weeks) also reported of discomfort in terms of tightness and challenges of getting them on and off, and many required help from a spouse or partner.

Interestingly many patients reported saving their stockings for flying.

### Knowledge of VTE symptoms

*Inadequate knowledge of symptoms*: One-third of the interviewed patients were of the opinion that they would not recognise symptoms of a blood clot. The other two-thirds described vague symptoms relating to DVT with ‘pain in lower leg’ being the most cited symptom, with a few mentioning tenderness, soreness, redness and swelling. Patients' responses demonstrated a lack of awareness of potentially fatal PE and only two patients described symptoms of PE, describing it as ‘when the clot travels to the lungs’ and cited symptoms such as shortness of breath, and tachycardia.

I mean, I know there is a risk but I wouldn't know how to assess whether or not I was having a blood clot. *Female aged 63, ovarian cysts*

Well if you're gonna ask me now what are the symptoms I'm gonna say I can't remember. I think discolouration, and probably pain in your leg. I don't know if you get to the stage of passing out do you? *Male aged 63, hip replacement*

*Reaction in the event of symptoms*: All participants said they would seek medical attention if they thought they were having a blood clot, some opting to go to the general practitioner (GP) and others recognising it as a medical emergency.

I would, I don't know, it depends how bad it was I suppose. I might go straight to A and E or, go to the doctor. But I would know that it's urgent. *Male aged 79, knee replacement*

### Postdischarge support

*Perceived role of primary care*: The interviews explored the need for postdischarge support from GP/district nurse. Generally patients were of the opinion that it was not necessary to routinely activate GP involvement postdischarge, and many could not see the point and found it difficult to envision a role for primary care in this area. Patients reported that they had all coped fine with the current system and were empowered to contact their GP if they had any concerns. A few said they would find it reassuring just to know they were doing the right thing due to the long time period between discharge from surgery and follow-up consultations.

That's quite interesting actually, I think I never thought about in terms of the GP but the one thing I did sort of think about during the whole recovery process is you kind of sort of leave hospital and that's it until you have your check-up so, you do hit moments where I think you do wonder is everything going ok even if you've got no reason to be, to question it. Am I doing the right thing? Should I be doing this? Shouldn't I be doing that? And there isn't really anyone to call. So whether it's a visit from the GP that would be, a good idea or whether it's a district nurse or whether even it's a physio, I think in that period between sort of discharge and you follow up consultation I think, it's too long a gap without some, just reassurance really, nothing more than that. *Female aged 40, hip replacement revision*

### Perceived gaps in patient education

*Patient education and public awareness*: Patients would however value more education on VTE, in terms of how the VTE prophylaxis works, clarity on stockings use and some information on symptoms in order to recognise if they were having a blood clot. One patient who had experienced a minor bleeding episode said it would have been useful to have been warned about possible side effects of pharmacological prophylaxis. Patients also touched on lack of public awareness of VTE and suggestions to deal with this included public campaign.

Y'know there's the sort of checklist that they have to fill before you're discharged. Y'know have you got your meds? Have you got this have you got that? Maybe that's the point at which they just need to sit with patients again and say, ‘right, let's just remember some key things y'know, do you know how to identify infection or blood clot?’…Y'know take an extra ten minutes per patient on discharge to go through a number of key risk areas is probably the one thing that could be, looked at. *Female aged 40, hip replacement revision*

## Discussion

The findings of this qualitative study give insights into patients' experiences of VTE prevention. Despite an awareness of VTE risk, patients did not appear to have a good understanding of the components of VTE and its potentially fatal complications. Patients were aware it was important to seek medical advice if they thought they had a VTE but appeared to lack the knowledge to assess its onset and important knowledge on signs and symptoms of VTE was limited.

Though our study found good adherence to injectable prophylaxis, there was poor adherence to antiembolism stockings due perhaps to a lack of clarity in patient education.

The National Institute for Health and Care Excellence (NICE) guidelines for VTE prevention recommend that patients discharged with VTE prophylaxis are offered verbal and written information on the signs and symptoms of DVT and PE, and the correct and recommended duration of use of VTE prophylaxis at home. It also recommends patients are educated about the importance of using VTE prophylaxis correctly, adherence and the importance of seeking help if they have any problems using the prophylaxis or DVT, PE or other adverse events are suspected.^2^ Our findings are inconsistent with these recommendations with patients having huge gaps in knowledge of symptoms and requirements relating to stockings use.

The finding relating to high adherence to injectable prophylaxis must be interpreted within the context of the study sample which was predominantly orthopaedic patients who had presurgical a session involving education on VTE prevention. Similarly this accentuates the significance of the finding relating to limited knowledge on signs and symptoms of VTE.

The literature supports the premise that improving a patient's understanding of the rationale for a medication increases adherence,^17^ and this has been proven to apply to VTE prevention—a US study found individualised patient education sessions on thromboprophylaxis was associated with higher adherence to injectable prophylaxis.^18^ Another study found that discharge counselling was associated with improved adherence after hospital discharge for myocardial infarction.^19^

### Strengths and limitations

To the best of our knowledge, this is the first qualitative study to explore hospitalised UK patients' perceptions and experiences of VTE prevention, incorporating awareness of VTE risk and VTE prophylaxis. Face-to-face interviews provided in-depth exploration of the issues, and analysis was iterative and independent of the interviewing researcher. The research team was multidisciplinary and offered different perspectives which enhanced interpretation of the data.

Surgical patients were over-represented, and inclusion of medical patients would have provided a broader representation of hospitalised patients; however, this was a feature of the composition of the survey respondents and findings have been interpreted in the context of this. The sample was also predominantly of white British ethnicity. Nevertheless, a maximum variety sample^20^ allowed a mix of participants from the sampling frame, and interviews were conducted to the point of theoretical saturation. Given the high proportion of orthopaedic surgical patients who often have VTE education embedded into the presurgical assessments, findings may overestimate the VTE awareness and adherence to VTE prophylaxis.

### Comparison with existing literature

Patients in our study had inadequate knowledge of symptoms of DVT and PE to enable appropriate self-assessment and self-reporting of possible VTE episodes. In addition, they did not appear to recognise the real personal risk of VTE.

Our finding that patients lack a clear understanding of VTE is consistent with previous research; a recent US study found that while hospitalised patients were aware of risk of VTE following orthopaedic surgery and the benefits of VTE prophylaxis many did not have a clear understanding of VTE.^6^ A survey in Canada found that only 6% patients who had received thromboprophylaxis as part of a hospital stay were aware of the complication of a blood clot travelling to the lungs, and 20% were not able to correctly identify a single symptom of DVT.^21^ The communication of risk is a difficult part of clinical practice^22^ and evidence suggests that the format in which risk information is presented affects patients’ understanding and perception of risk.^23^ Some areas of risk communication still lack strong evidence,^23^ nevertheless the communication of VTE risk should aim to influence patient awareness of VTE and correct inappropriate risk perception to facilitate patients to reduce their risk. It is therefore important for patients to be aware that they have been assessed as high risk of VTE. The literature also suggests inadequate public knowledge of VTE and a recent UK street survey reported limited public knowledge of DVT and highlighted the need for raising general awareness of DVT with particular focus on its complications.^24^

Our finding relating to injectable prophylaxis is contrary to the literature which indicates suboptimal adherence to heparin injections^7^ ^8^ with non-adherence ranging from 21% to 37%.^7^ Our study also found that patients often did not receive enough information to support proper use of elastic compression stockings resulting in poor adherence. This is consistent with the literature and other researchers have observed that some inpatients are offered stockings in a perfunctory manner with poor or limited patient education on VTE.^25^ ^26^

Provider–patient communication in hospitals is frequently problematic and often further complicated during hospital discharge.^27^ ^28^ Incorporating the patient's perspective enriches and improves communication between providers and patients, and integrating collaboration and patient empowerment has positive outcomes in relation to patient satisfaction and healthcare outcomes.^29^

## Conclusion

This study addresses an important aspect of VTE prevention and identifies gaps in patient education that hinder patients' role in VTE prevention. While some patients are aware of the appropriate use of pharmacological and mechanical prophylaxis postdischarge, many lacked important knowledge on the use of antiembolism stockings and symptom recognition of DVT and PE. Patients need a basic but comprehensive understanding of VTE and appropriate use of VTE prophylaxis to complete their participation in VTE prevention.

Suboptimal adherence to VTE prophylaxis and the lack of awareness of VTE symptoms compromise VTE prevention and puts patients at risk of adverse events; therefore, more attention must be paid to patient involvement in VTE prevention. Improved patient education incorporating VTE risk will motivate adherence to VTE prophylaxis, and education on recognition of symptoms will equip patients to self-assess and self-report possible VTE events. Ongoing initiatives such as Thrombosis UK and World Thrombosis Day may help to increase awareness and improve the understanding of venous thrombosis. Nevertheless, patient education must be systematic and standardised across the National Health Service (NHS) to optimise the effectiveness of the national VTE prevention strategy.
