# Supplementary material for: Patients' perceptions and experiences of the prevention of hospital-acquired thrombosis: a qualitative study
Source: BMJ Open. 2016 Dec 13;6(12):e013839. doi: 10.1136/bmjopen-2016-013839 (PMC5168621; doi:10.1136/bmjopen-2016-013839)
Supplement: supplementary appendix [file bmjopen-2016-013839supp_appendix1.pdf]

Appendix 1. Interview topic guide

General

1. Were you aware of a risk of blood clots associated with your condition (or being in hospital) before you went into hospital?
2. Were you aware of being risk assessed for blood clots? *Prompt: Can you tell me about your assessment and what you were told?*
3. Can you explain to me why you needed treatment for blood clots?
4. What information did you receive about blood clots?
5. Was the information you were given clear to understand?
6. How satisfied were you with the information you received?
7. How could the information be improved?
8. Do you feel the information was given to you at the right time during your procedure/ hospital stay? *Prompt: If not, when would have been better a better time?*
9. What blood clot treatment did you receive whilst you were in hospital?
10. What blood clot treatment did you receive when you were sent home from hospital?
11. Did you have any contact with any health professionals after you left the hospital? *Prompt: Did they talk to you about blood clots/your treatment?*
12. Would you have benefited from contact with a health professional (i.e. a visit from a district nurse)?
13. Do you think it would have been useful to see your GP? *Prompts: What help would you have liked from your GP? At what stage would it have been helpful to see your GP regarding blood clots… being going into hospital /after leaving the hospital?*

I would now like to ask you about the particular treatment you received…

Stockings

14. What information/advice did you receive about surgical stockings?
15. Did you understand why you needed to wear surgical stockings?
16. How long were you advised to wear surgical stockings? *Prompts: Was this explained to you fully? How long did you wear them? Please expand ....*

Injections

17. What information/advice did you receive about injections to prevent blood clots?

18. Did you understand why you needed to have injections?

19. Did you give yourself the injections? *Prompts: How did you feel about giving yourself injections? Did you feel you received sufficient training to give yourself injections? Would you have liked more practice in giving yourself the injections?*

20. Were you given the option for a health professional to help with the injections?

21. Did you complete your course of treatment? Please expand.....

Other treatment

22. What information/advice did you receive about your treatment to prevent blood clots?

23. Did you understand why you needed that particular treatment?

24. Did you feel you could manage the treatment you were given?

25. Did you complete your course of treatment? Please expand.....

Understanding, knowledge and awareness

26. Reflecting on your experience how could your understanding of blood clots and treatments have been improved?

27. Do you feel that you know enough about the risks of blood clots now?

28. Would you be able to recognise the symptoms of having a blood clot?

29. Who would you contact for advice on blood clots now?

30. Who would you contact if you thought you had a blood clot?

31. Blood clots are a big problem associated with being in hospital. What do you think could and should be done to increase awareness for the general public?
