# Supplementary material for: Patients' perceptions and experiences of the prevention of hospital-acquired thrombosis: a qualitative study
Source: BMJ Open. 2016 Dec 13;6(12):e013839. doi: 10.1136/bmjopen-2016-013839 (PMC5168621; doi:10.1136/bmjopen-2016-013839)
Supplement: supplementary appendix [file bmjopen-2016-013839supp_appendix2.pdf]

Appendix 2. Section of Nvivo report for the theme: experiences of elastic compression stockings

Notes:

The individual Nvivo reports comprised of all interview data relating to the theme for each individual participant. The Nvivo report for each theme was reviewed to identify patterns in the data which were then pieced together to give a complete description of the subject.

--

<Internals\\P04_NOC099> - § 1 reference coded [6.51% Coverage]

Reference 1 - 6.51% Coverage

Q: So, you didn't experience were we have to, wear surgical stockings or anything like that?

A: Well I did, oh yes.

Q: Did you have surgical stockings?

A: Oh yes, I had those for the six weeks. Yeah.

Q: And you wore them for the six weeks did you?

A: Oh yes. Yes. I always do everything as I'm told because there's absolutely no point in, consulting somebody and then disagreeing with them.

Q: Oh that's good, people often think, oh I'll wear it for a couple of weeks and that's it.

A: No, every day.

Q: And did they train you to wear them, or how to put them on?

A: I have a wife for that.

Q: So you did, oh. So your wife [inaudible <1 sec] 11:46

A: Well I got my own back 'cause I had to do hers so, she had to mine. They were difficult for her, because with her limp, and everything and her back, not sort of back problem except slightly weak she find it quite difficult to do the stockings. OK got used to it in the end and, yeah they were on and off, but it was difficult, and that was an area which I think somehow that can be improved, how anybody on their own does it, I don't know. You cannot do it on your own, and however much you've got this, the gadget, and I've got the [inaudible <1 sec] 12:21 one, the socks, which fantast – I'm still using today. Fantastic, the smooth one, it's not easy. Although stockings are supposed to be tight so…

<Internals\\P05_NOC035> - § 3 references coded [20.75% Coverage]

Reference 1 - 9.96% Coverage

A: As far as I'm aware, there was, I think, there were injections, I don't think there were tablets as well but I could be wrong but the injections which then carried on, for four weeks once I'd been discharged and the stockings.

Q: How long did you have to wear the stockings?

A: I suppose that's probably the most unclear part of the whole procedure, the injections were fine, I was quite happy with, y'know, doing and administering it and that whole process, obviously the stockings were worn in the hospital sort of continuously and then I think the information you get, the information sheets is wear of up to four weeks post op, but there was definitely conflicting advice from the nursing staff. Some of the nurses were, 'well it's not that serious if you don't wear them' others were 'absolutely must wear them.' So, y'know there was definitely conflicting advice.

Q: Oh, that's interesting.

A: From the nursing staff on the stockings and to be honest, I found then incredibly irritating. So I tended to – I wore it for the first week and then I just scrapped it, and I thought well, I've got the injections anyway, I am quite, I was quite ac – so what I did, my view was, I won't wear the stockings but what I'll do is I'll move a lot more, so y'know even if I was in bed I was sort of doing leg exercises and keeping movement going there, I kind of thought I'd rather do more of that, than wear the stockings, now whether or not that's, advisable who knows but, I can only apply my own logic.

Q: They sound as though they're really uncomfortable.

A: I think it's not so much that they're uncomfortable, as that they really start to itch after a while. And, it's that and either you, that kind of person would not and I just found the itching got on my nerves so I just, couldn't be bothered after that.

Reference 2 - 3.89% Coverage

Q: Did you feel that you had enough information about the blood clots?

A: Yeah, I think so, I do think so, I think, maybe just, I think maybe clarity on the stockings and how important those are is maybe necessary but again that might just be down to individual experiences I don't know. I think what's also sort of true to say y'know if you weren't, if I had been unsure I think, the staff and ward then approachable if you had extra questions or you weren't sure so I think, yeah you could've y'know, if I wasn't clear I don't think there would've been, a problem asking. Even the nursing staff or the surgeon or whoever, I think they would've been more than happy to re-explain it, but I was alright with it.

Reference 3 - 6.90% Coverage

Q: If you went into the hospital again do you think you know enough now about the risks of the blood clot?

A: Probably, yeah.

Q: And do you think that would make you feel more able to ask for information?

A:	Yeah, I suppose so. I mean I would certainly, if I went in now, I would certainly ask more questions, for example about the stockings, and y'know. And I would probably ask the surgeon rather than the nursing staff.

Q:	Why do you say that?

A:	Just because I think the nursing staff have, different views on it, I mean it was interesting 'cause what the nursing staff actually said is that they were just in the process of changing the protocols, for hip and knee replacements in terms of treatment and nursing protocols, so there seems to be quite a lot of difference in nursing staff that were adopting new protocols versus those who were still applying old protocols so it did seem a bit of inconsistency in the way the nursing staff were dealing with it but I don't think it was their fault, I think it was policy really. So… everyone y'know there were some who were more probably tied to older ways of doing things and were going to stick to that. Y'know, and I mean it's certainly no criticism of the nursing staff I just think it's, y'know.

<Internals\\P06_NOC100> - § 1 reference coded [8.80% Coverage]

Reference 1 - 8.80% Coverage

Q:	So you wore you surgical stockings for the full time.

A:	Yes, I did yes. I—

Q:	And how did you find that?

A:	Oh I hated those, hated them. I found that the design of them was awful because they had this little hole in the bottom, which, at night got trapped around your toes, so you'd wake up and your toes were freezing because they'd been stopping the circulation, and then in the daytime when you were wearing shoes to go out, they trap around your toes as well, they'd work up, no matter what we did, and of course my husband had a terrible job getting them on…{*potentially identifiable text removed*}

Q:	Oh right!

[both laugh]

A:	And my friend is patient B has had a different one, and a different hospital y'know, but, anyway, no the stockings, I found very very uncomfortable and, very hot but I mean I knew I had to keep using them, and I did for the ti—well five weeks until I saw [person 1], and then I said to him 'please can I leave these things off' and he looked at my leg and said 'you've got not swelling at all' he said 'it's amazing,' I said nothing 'certainly,' he said 'just keep walking an mobile and', y'know which I've done and, he said 'yes you can take them off' 'Oh,' I said 'thank goodness,' I said 'you can have them here and now so there you go.

Q:	How did you find them, could you put them on and off on your own?

A:	No.

Q:	No.

A: No, I couldn't. Well the problem is I've got a back problem, and I can't bend right down anyway to this leg, this one I can but not this one I, not been able to do that for years, so I couldn't put them on at all they were just too tight and I mean [person 2] had a job to get them on to they were really tight, but, my friend who had a hip replacement, she had them where they, the toes were completely free, and then they had a hole for the heel. And that caused it's own problems that she got a sore heel, but if you raise your feet a little bit, y'know it helps but, no, I can't say they were wonderful. I'd like another design on those, y'know.

<Internals\\P07_NOC046> - § 1 reference coded [26.55% Coverage]

Reference 1 - 26.55% Coverage

Q: What about the surgical stockings, what information did they give you about wearing those?

A: Just basically they had to be worn, y'know, because of the clots basically. So yeah, yeah, knew that I had to wear them, yeah.

Q: Yeah, and you were quite happy with the information that they gave you.

A: Yeah, it was all good, yeah, very good, yeah. I couldn't, I couldn't be more pleased with [place 1], to be honest with you, [hospital 1], absolutely superb. Really do. Yeah, really good.

Q: Yeah, everybody has said that. Did you wear the stockings all of time?

A: For the full length I was in there, yeah.

Q: And when you came out?

A: Didn't wear them when I came out.

Q: Were you supposed to?

A: Well no I don't think so, because, I wasn't really sitting around as such, once I got out I was on my crutches and I was, trying to get out and about, didn't really feel up but I was, I was walking again within a week, so I was sort of up and about more, so I wasn't as if I was just lying still for, yeah, so I didn't wear them when I came out no, no. Although they did give me four or five pairs.

Q: Do you think perhaps they'd intended you to wear them?

A: Yeah, probably.

Q: So why did you not?

A: I don't know really. I just think 'cause I was out, up and about and, feeling y'know, that I'm moving so everything is moving sort of thing so, yeah.

Q: And how did you find them when you were wearing them?

A: Fine. Yeah, absolutely fine yeah.

Q: Did the nurses take them on and off for you?

A: Yeah, the, yeah they came and measured me in the morning, with the right size and, yeah they come and put them on, yeah. Oh what was it, the leg, this leg, I had the operation on this time, they did the, they froze it, because I have a bit of an allergic reaction to the anaesthetic, makes you really sick, so they didn't give me so much anaesthetic and they did a something in the leg, I couldn't feel my leg for about three days, obviously I didn't have one on the leg that that was done, this leg, yeah, but no everything was yeah it was all fine.

<Internals\\P08_NOC110> - § 2 references coded [13.99% Coverage]

Reference 1 - 10.71% Coverage

A: That's right. The beasting type, to call it. But, I assumed that it was in the medication I didn't, and of course the stockings because it's,

Q: You wore those in hospital.

A: I wore those in hospital.

A: That's right. And it was.

A: Yes it was always a bit of a joke, having to get them on and off, y'know if I wanted to shower or something.

Q: Did you manage to do that on your own?

A: Never managed to do it, now I've got short arms unfortunately, and of course if you've got hip problems, having had both my hips I mean they're, that one's only ninety percent flex, and this one obviously just been done, so it's a bit difficult to do that, so I had to have help with those.

Q: Who helped you?

A: Well in that case the nurse did. Yeah, in hospital the nurse helped me. When I came home of course my wife helped me because she's, used to it. Yes, but it is virtually impossible to put them on by yourself. I have got two friends who've had hip ops and they've both had to get the nurse out to do it, yeah. 'Cause they didn't have a wife. It's a…

Q: Very much people rely on their wives or spouse.
A: Well we do if we're lucky enough to have them, yes! But of course a lot of people don't. Well you can get up, we're lucky in this village you see because we've got our own medical centre. So we can go up there and they're very accommodating, they'll do anything yeah. So that's great.

Q: So you were quite fully aware of why you need to wear them.

A: Yes, absolutely yes.

Q: So when you came out, was it just the stockings?

Reference 2 - 3.28% Coverage

Q: And you wore stockings as well. And how long did you wear those for?

A: Well I had, I had them until I got, I had to go down to see [surgeon 1] who's the surgeon, and I wore them right up to that time which was six weeks.

Q: Six weeks. And you were OK with that then.

A: No problem.

Q: They're quite uncomfortable the stockings?

A: Yes they are but, not so bad in the winter.

Q: [laughs] oh yes!

A: In the summer you get all sticky. Quite uncomfortable.

<Internals\\P11_NOC053> - § 1 reference coded [21.56% Coverage]

Reference 1 - 21.56% Coverage

Q: Did you wear surgical stockings?

A: In hospital yes. Not afterwards though.

Q: That's—oh I see—

A: I did take them out but I didn't wear them.

Q: Oh, were you supposed to then?

A: I wasn't told to.

Q: But they gave you some to take out?

A: Well I think you just, keep hold of them because they're manky.

Q: Just keep the one's you've got.

A: Use them for flying when I go on holiday

[both laugh]

Q: Yeah, why not.

A: I don't know, I suppose you're still sitting around when you're, I mean the first week I lay on the sofa but you're up and down a little bit more than you were in the hospital. But no I didn't wear them when I came home.

Q: So you weren't told that you should or,

A: No.

Q: You understood why you wore them in the hospital?

A: Yeah, yeah.

The only thing that was really bad chilblains on that foot. On that—only on that foot, ‘cause it was obviously winter when I was—and they said it was ‘cause I was laying still so I suppose, if I was laying still enough to get chilblains and get cold, I maybe should’ve had—but I wasn’t told so I didn’t do it.

Q: That’s interesting.

A: They’re not very nice, you just take them off as soon as you get home.

Q: So had you been told that you should’ve worn them?

A: I would’ve. Yeah, yeah.

Q: I wonder whether you should’ve worn them for some time afterwards.

A: Maybe a couple of weeks, yeah.

Q: Had—do you think perhaps you might not have had the right instructions and, would you have worn them had you have been, told.

A: Well I would’ve but I don’t know what the policy is. And so… I don’t know.

A: I would say I wore them for, I think I went back on the tenth of January which was approximately six weeks, I wore them for five and a half weeks, yes.

Q: Yes, so you did, yes. a lot of people find them so uncomfortable

A: Well, they were, I mean once they go on I think you forget about them and, as I say I probably well, I was, towards the end I was probably showering most days, well I don’t know, I think if I had shower, perhaps I was showering every other day at the time, [inaudible <1 sec] 4:56, so I would change them, every other day. [Inaudible 1 sec] 5:00

<Internals\\P15_ORH008> - § 2 references coded [17.03% Coverage]

Reference 1 - 6.04% Coverage

A: That was the pre-op. So, it’s whether you read it or not so it’s very hit or miss whether anybody takes any notice of it because as I say, we’re given so much information. When I actually then went in to have my surgery, it was just one of the health care assistants that gave me my gown and, gave me the stockings to—saying to put them on but didn’t explain anything about them. But I’m a nurse so, I know.

Q: Did she know you were a nurse?

A: No, no.

Q: Did—

A: No, but they were just—they weren't there, they were just there to sort of hand out, you need to put your gown on, you need to put these socks on, I'll come back and help you put them on if you want and, that was it there was nothing, I suppose I could've asked, what are they for but I knew, and I had read the leaflets. Which—

Reference 2 - 11.00% Coverage

: No , You've got your priorities haven't you, if you're going for an operation your priority is your operation, not thinking about something else that might happen to you. I can appreciate that. So they didn't actually explain to you why you needed to wear surgical stockings,

A: No.

Q: Did you have to wear them after you came out of hospital?

A: No.

Q: Did you just stop—did they take them off you when you left?

A: No they didn't, I brought them home and I've actually got them at home 'cause I thought they might come in handy if I went on a plane.

Q: Yes.

A: So actually, they're in the wash basket, I never got round to washing them since the November, they sat there waiting for a day when I might think, oh I'll wash those and, put them in a drawer so…No, and I didn't, I was a day case, and, they said I didn't need any injections or anything. [Inaudible <1 sec] 4:52

Q: Oh you, so you didn't have any injections [inaudible <1 sec] 4:54, no.

A: No, I didn't have anything at all, just that sock.

Q: —they didn't say to you, you need to wear these for so long afterwards.

A: No no I didn't wear them. 'Cause I was up and about.

Q: Yeah, no. I mean, most people say that, if they feel they're getting enough movement then they do justify not wearing them because they're very uncomfortable.

A: I wasn't told that I should've worn them, I mean

Q: you didn't have any injections either?

A: No.

--
